# Comprehensive measurement of the prevalence of dementia in low- and middle-income countries: STRiDE methodology and its application in Indonesia and South Africa

**DOI:** 10.1192/bjo.2023.76

**Published:** 2023-06-06

**Authors:** Nicolas Farina, Roxanne Jacobs, Yuda Turana, Fasihah Irfani Fitri, Marguerite Schneider, Imelda Theresia, Sumaiyah Docrat, Tara Puspitarini Sani, Lydia Augustina, Emiliano Albanese, Adelina Comas-Herrera, Petra Du Toit, Cleusa P. Ferri, Ishtar Govia, Aliaa Ibnidris, Martin Knapp, Sube Banerjee

**Affiliations:** Centre for Dementia Studies, Brighton and Sussex Medical School, UK; and Community and Primary Care Research Group, University of Plymouth, UK; Alan J Flisher Centre for Public Mental Health, University of Cape Town, South Africa; Faculty of Medicine and Health Sciences, Atma Jaya Catholic University of Indonesia, Indonesia; Department of Neurology, University of Sumatera Utara, Indonesia; Alzheimer's Indonesia, Indonesia; Institute of Public Health, Faculty of Biomedical Sciences, Università della Svizzera Italiana, Switzerland; Care Policy and Evaluation Centre, London School of Economics and Political Science, UK; Alzheimer's South Africa, South Africa; Psychogeriatric Unit, Department of Psychiatry, Escola Paulista de Medicina, Universidade Federal de São Paulo, Brazil; Epidemiology Research Unit, Caribbean Institute for Health Research (CAIHR), The University of the West Indies, Jamaica; Faculty of Health, University of Plymouth, UK

**Keywords:** Dementia, low- and middle-income countries, epidemiology, outcome studies, statistical methodology

## Abstract

**Background:**

A core element of the Strengthening Responses to Dementia in Developing Countries (STRiDE) programme was to generate novel data on the prevalence, cost and impact of dementia in low- and middle-income countries, to build better health policy. Indonesia and South Africa are two middle-income countries in need of such data.

**Aims:**

To present the STRiDE methodology and generate estimates of dementia prevalence in Indonesia and South Africa.

**Method:**

We conducted community-based, single-phase, cross-sectional studies in Indonesia and South Africa, randomly sampling participants aged 65 years or older in each country. Dementia prevalence rates for each country were generated by using the 10/66 short schedule and applying its diagnostic algorithm. Weighted estimates were calculated with national sociodemographic data.

**Results:**

Data were collected between September and December 2021 in 2110 people in Indonesia and 408 people in South Africa. The adjusted weighted dementia prevalence was 27.9% (95% CI 25.2–28.9) in Indonesia and 12.5% (95% CI 9.5–16.0) in South Africa. Our results indicate that there could be >4.2 million people in Indonesia and >450 000 people in South Africa who have dementia. Only five participants (0.2%) in Indonesia and two (0.5%) in South Africa had been previously diagnosed with dementia.

**Conclusions:**

Despite prevalence estimates being high, formal diagnosis rates of dementia were very low across both countries (<1%). Further STRiDE investigations will provide indications of the impact and costs of dementia in these countries, but our results provide evidence that dementia needs to be prioritised within national health and social care policy agendas.

There are currently an estimated 50 million people with dementia worldwide, with this expected to rise to 152.8 million by 2050.^1^ The growth in numbers of people with dementia is largely driven by increasing life expectancy in low- and middle-income countries (LMICs). However, many LMICs lack basic national prevalence data on dementia, and so are reliant on estimates based on regional statistical modelling, as used within the Global Burden of Disease study.^1^ These estimates, although useful for their global coverage, are limited by the robustness of the model and the availability of country-specific data. They are also less powerful than local data in making the case for national policy priority. The Strengthening Responses to Dementia in Developing Countries (STRiDE) programme identified that policy makers and key stakeholders wanted robust national estimates of dementia prevalence, and that there was a reluctance to act on data derived even from geographically close or socioeconomically similar settings.^2^ In two STRiDE countries, South Africa and Indonesia, local prevalence data were identified as a priority need.^3^

In South Africa, there are few studies that explore dementia prevalence. The single best evidence comes from 1394 Xhosa-speaking older adults in Cape Town.^4^ The study used a dementia screening tool, the Brief Community Screening Instrument for Dementia (CSI-D),^5^ and estimated dementia prevalence to be 11% for those aged 65 years and older.^4^ Other estimates of dementia prevalence come from studies with small sample sizes and potentially non-representative samples.^6^ Evidence on dementia prevalence from Indonesia is geographically limited to the islands of Java and Bali.^7–9^ Excluding issues of generalisability, these studies have often reported unusually high prevalence estimates (>20%) compared with many international estimates (e.g. 4–9%, aged 60 years and older).^10^

## Aims

STRiDE aimed to develop and deliver a pragmatic methodology to generate accurate dementia prevalence estimates in LMICs, sampling from rural and urban areas, using South Africa and Indonesia as exemplars. This methodology seeks to improve on existing evidence by minimising internal and external bias, and simultaneously generating data to measure both the impact and cost of dementia in these two settings, and with appropriate cultural adaptation in other LMICs.

## Method

The STRiDE programme developed a common data collection approach with the capacity for methods to vary pragmatically to access and use existing sampling frames.

### Participants

Recruitment occurred in two sites in each country: Jakarta and North Sumatra in Indonesia, and Limpopo and Western Cape in South Africa. Sites were selected for pragmatic reasons and to ensure heterogeneity in terms of socioeconomic status and rurality. Random sampling was used: simple randomisation in Limpopo and proportionate to population size randomisation in other sites. Details of sites and sampling strategy are shown in Supplementary Appendix 1, available at https://doi.org/10.1192/bjo.2023.76.

To be eligible, participants were required to be aged 65 years or older at the date of consent, speak one of the languages of the adapted toolkits (Afrikaans, Bahasa Indonesian, English, isiXhosa, Sepedi) and live within the defined sampling areas. We checked the age of participants informally before consent and more rigorously following consent (e.g. from official documents and using the calendar method). All participants were required to identify an informant who could provide supplementary information. The informant could be anyone with a close relationship with the older adult and who spoke the appropriate language. Potential participants were excluded if they resided in care or nursing homes, or they lacked capacity to consent and could not identify a personal consultee to assist in the consent process.

### Procedure

Researchers visited potential participants’ homes (or another location convenient to participants) in pairs. Informed consent was obtained (written or oral) from the older adult and an identified informant. Researchers initially completed a core set of questions related to age and household with both the informant and older adult. Subsequently, the older adult and informant completed the remaining questionnaires independently of each other, one with each of the researchers. Measures pertaining to the identification of dementia (as described below) were prioritised. In a single-stage process, all participants were asked the same set of questions, with the exception of some branching (e.g. care-related questions were skipped if no care was provided). Study data were collected and managed with REDCap electronic data capture tools (REDCap Consortium, Vanderbilt University, Nashville, TN, USA; https://projectredcap.org/) hosted at the London School of Economics and Political Sciences.^11^

All researchers were provided standardised training in how to administer the questionnaires before testing. We developed a series of presentations and standard operating procedures centrally to guide researchers. Data collection occurred between September 2021 and December 2021.

### Measures

A series of demographic measures were collected, including age (ascertained through a hierarchy of self-report, informant report, official documentation and the calendar method), sex, literacy (ability to read and write) and self-report receipt of a diagnosis of dementia or Alzheimer's disease. The following instruments were completed: 10/66 short schedule, Dementia Severity Rating Scale (DSRS) and Lawton Activities of Daily Living Scale.

The 10/66 Short Dementia Diagnostic Schedule^12^ is composed of the following measures: (a) the CSI-D, a screening instrument for dementia for use in cross-cultural studies,^13^ with both a cognitive assessment component and an informant-reported functional impairment component; (b) the EURO-D, a self-report measure to screen for depression;^14^ and (c) the Consortium to Establish a Registry for Alzheimer's Disease (CERAD), a ten-word list learning task with delayed recall.^15^ We used the 10/66 short algorithm to generate an estimate of dementia caseness,^12^ which uses data derived from the CSI-D, CERAD word list and EURO-D (Supplementary Appendix 2). The 10/66 short algorithm has been demonstrated to have good sensitivity across multiple settings,^12^ including against clinical diagnosis in Singapore (area under the curve (AUC) = 0.87),^16^ Switzerland (AUC = 0.74)^17^ and Pakistan (AUC = 0.85).^18^

The DSRS is a brief informant report measure of 12 functional abilities similar to those in the Clinical Dementia Rating (CDR) scale.^19^ The DSRS predicts the CDR sum of boxes score.^20^ Scores range from 0 to 54, with higher scores representing greater impairment. The measure was completed as an informant report measure.

The Lawton Activities of Daily Living Scale is a short questionnaire that covers eight instrumental activities of daily living.^21^ The measure was completed as an informant report measure.

Measures of cost and impact were also completed, but these did not contribute to the dementia prevalence calculations and are not reported here. Indonesian participants were interviewed in Bahasa Indonesian, and South Africa participants were interviewed in isiXhosa, Sepedi, Afrikaans or English. Details of the full STRiDE toolkit and the underlying cross-cultural adaptation and translation process are described elsewhere.^3^

### Ethics

The authors assert that all procedures contributing to this work comply with the ethical standards of the relevant national and institutional committees on human experimentation and with the Helsinki Declaration of 1975, as revised in 2008. All procedures involving human patients were approved by the London School of Economics and Political Sciences, University of Cape Town, University of Sumatera Utara (approval number 862/KEP/USU/2020) and Atma Jaya Catholic University (approval number 01/12/KEP-FKIKUAJ/2020).

### Sample size calculation

Precision calculations indicated that an overall sample of 2039 would allow the estimation of an expected dementia prevalence of 4.5% with a precision of ±0.9% within each country. The recruitment target was increased to 2200 to allow for missing data.

### Analysis

Demographic data were generated separately for each country; we present key demographics for dementia occurrence and assessment (age in 5-year intervals, sex, literacy), in line with previous dementia prevalence research.^22^ We investigated representativeness of the study sample in a series of Pearson's chi-squared analyses, which were used to ascertain whether demographic factors differed between those with complete or missing data (i.e. those for which we had sufficient data to run the diagnostic algorithm).

We calculated total prevalence (10/66 short algorithm) estimates unweighted, with 95% confidence intervals. We then weighted data by national demography (age, sex and literacy), and computed national proportions from Indonesia and from South Africa. We generated weights based on sequential computation (Supplementary Appendix 3).

Next, we ran logistic regression models to explore factors potentially associated with increased risk of dementia, and subsequently calculated age-adjusted odds ratios for sex and literacy. We also ran supplementary Poisson regression models to generate prevalence ratios. We explored convergent validity of the 10/66 short algorithm against cognitive impairment (Brief CSI-D screening tool cognitive scale), functional impairment (Brief CSI-D screening tool informant scale, Lawton Activities of Daily Living scale) and care needs (older adult needs care, yes/no), reporting the effect size between populations (Hedges’ *g*). For Hedges’ *g,* a value >0.5 indicates a medium effect size, and a value >0.8 indicates a large effect size. In addition, participants who scored positive for dementia on the 10/66 short algorithm were compared against existing cut-offs of dementia on the DSRS and the Brief CSI-D (Supplementary Appendix 4). For these comparisons, we calculated the AUC ((sensitivity + specificity)/2) between measures. We interpreted the AUC according to existing criteria: a score of 0.5–0.6 is considered poor accuracy, 0.6–0.7 is considered acceptable accuracy, 0.7–0.8 is considered good accuracy and >0.8 is considered very good or excellent accuracy.^23^

## Results

**Fig. 1:**
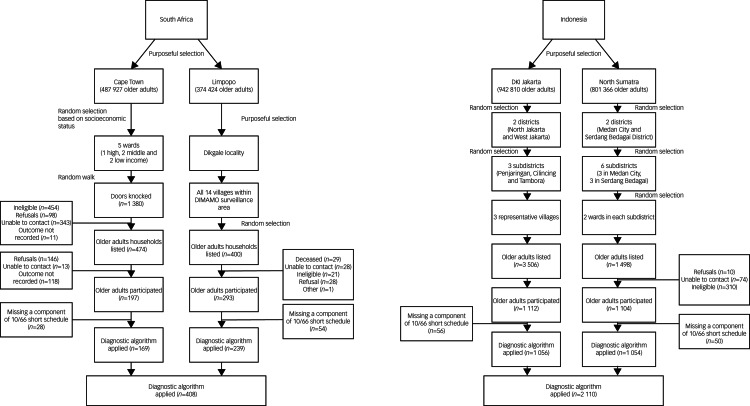
Participant recruitment flow diagram within each site, for September to December 2021. The 10/66 short schedule refers to the 10/66 Short Dementia Diagnostic Schedule. DIMAMO, Dikgale, Mamabolo and Mothiba; DKI, Daerah Khusus Ibukota.

See Fig. 1 for a flow diagram of participant recruitment. In Indonesia, we recruited to target; in South Africa, we adhered to the planned recruitment strategy and procedures, but we were unable to reach the target sample size because of disruptions caused by the COVID-19 pandemic.

### Missing data

In Indonesia, we recruited 2216 participants. In South Africa, we recruited 490 participants. Across sites, there were instances in which only partial data were available because of participant refusal, or researcher or technical error. In the Indonesian cohort, there were 106 participants (4.8%) with insufficient data to run the 10/66 short algorithm. Missing data were not associated with age (*n* = 2216, *χ*^2^ = 2.64, *P* = 0.76), literacy (*n* = 2173, *χ*^2^ = 0.37, *P* = 0.54) or sex (*n* = 2216, *χ*^2^ = 0.88, *P* = 0.35). In the South African cohort, there were 82 participants (16.7%) for whom we were unable to run the 10/66 short algorithm, predominantly because of refusal to answer the EURO-D (*n* = 64). Ability to run the algorithm was not associated with age (*n* = 489, *χ*^2^ = 4.04, *P* = 0.54), literacy (*n* = 470, *χ*^2^ = 0.33, *P* = 0.56) or sex (*n* = 467, *χ*^2^ = 0.01, *P* = 0.92).

### Demographics

Dementia prevalence was estimated in 2110 older adults in Indonesia and 408 older adults in South Africa. Mean age of participants was 71.1 (s.d. = 5.42) years in Indonesia and 74.8 (s.d. = 7.42) years in South Africa. Both country samples contained higher proportions of women than men (up to 63.5% in South Africa) (Table 1). Men were 2.50 times more likely to be literate in Indonesia than women (Mantel–Haenszel *χ*^2^ = 49.66, *P* < 0.001). Men were 1.73 times more likely to be literate in South Africa than women (Mantel–Haenszel *χ*^2^ = 4.71, *P* = 0.03). See Supplementary Appendix 5 for the number of participants by country, age, sex and literacy.

**Table 1:** Summary of key demographic variables, split by country

	Indonesia (*N* = 2110)		South Africa (*N* = 408)	
	Mean (s.d.)	*n* (%)	Mean (s.d.)	*n* (%)
Age, years	71.1 (5.43)		74.8 (7.42)	
Site				
Jakarta		1063 (50.4%)		Not applicable
North Sumatra		1047 (49.6%)		Not applicable
Cape Town		Not applicable		169 (41.4%)
Limpopo		Not applicable		239 (58.6%)
Sex				
Male		853 (40.4%)		133 (32.6%)
Female		1257 (59.6%)		259 (63.5%)
Missing		0 (0.0%)		16 (3.9%)
Literacy				
Illiterate		369 (17.5%)		95 (23.3%)
Literate		1710 (81.0%)		298 (73.0%)
Missing		31 (1.5%)		15 (3.7%)
Language				
Indonesian Bahasa		2110 (100.0%)		Not applicable
isiXhosa		Not applicable		51 (12.5%)
Sepedi		Not applicable		235 (57.6%)
Afrikaans		Not applicable		37 (9.1%)
English		Not applicable		85 (20.8%)
Missing		0 (0.0%)		0 (0.0%)
Relationship of informant				
Spouse		436 (20.7%)		87 (21.3%)
Son/daughter		1063 (50.4%)		152 (37.3%)
Son/daughter-in-law		175 (8.3%)		20 (4.9%)
Sibling		54 (2.6%)		5 (1.2%)
Other relative		58 (2.7%)		57 (14.0%)
Friend		1 (0.0%)		12 (2.9%)
Neighbour		81 (3.8%)		27 (6.6%)
Other		242 (11.5%)		47 (11.5%)
Missing		0 (0.0%)		1 (0.2%)

Only five participants (0.2%) in Indonesia and two (0.5%) participants in South Africa had been previously diagnosed with dementia.

### Prevalence

Unweighted estimates of dementia for those aged 65 years and older were 26.6% (95% CI 24.8–28.6) in Indonesia and 14.5% (95% CI 11.2–18.3) in South Africa. After national weighting, estimates marginally increased to 27.9% (95% CI 25.2–28.9) in Indonesia and decreased to 12.5% (95% CI 9.5–16.0) in South Africa. Unweighted prevalence estimates by country, age, sex and literacy are reported in Table 2.

**Table 2:** Prevalence estimates split by age, sex and literacy, using the 10/66 short-form algorithm (grand total prevalence is also reported in both weighted and unweighted formats)

	Indonesia			South Africa		
	10/66 short-form dementia diagnosis algorithm			10/66 short-form dementia diagnosis algorithm		
	Cohort, *n*	Positive cases, *n*	Positive cases, rate, % (95% CI)	Cohort, *n*	Positive cases, *n*	Positive cases, Rate, % (95% CI)
Male, age (years)						
65–69	443	74	16.7 (13.4–20.5)	45	1	2.2 (0.1–11.8)
70–74	245	47	19.2 (14.4–24.7)	40	6	15.0 (5.7–29.8)
75–79	110	35	31.8 (23.3–41.4)	23	1	4.3 (0.1–21.9)
80–84	40	19	47.5 (31.5–63.9)	12	3	25.0 (5.5–57.2)
85–89	13	2	15.4 (1.9–45.4)	10	6	60.0 (26.2–87.8)
≥90	2	0	0.0 (0.0–84.2)	3	1	33.3 (0.8–90.6)
Total	853	177	20.8 (18.1–23.6)	133	18	13.5 (8.2–20.5)
Female, age (years)						
65–69	574	134	23.3 (19.9–27.0)	70	1	1.4 (0.0–7.7)
70–74	367	103	28.1 (23.5–33.0)	69	9	13.0 (6.1–23.3)
75–79	182	70	38.5 (31.4–45.9)	45	6	13.3 (5.1–26.8)
80–84	97	51	52.6 (42.2–62.8)	39	7	17.9 (7.5–33.5)
85–89	27	19	70.4 (49.8–86.2)	25	8	32.0 (14.9–53.5)
≥90	10	8	80.0 (44.4–97.5)	11	4	36.4 (10.9–69.2)
Total	1257	385	30.6 (28.1–33.3)	259	35	13.5 (9.6–18.3)
Literacy^a^						
Total literate	1710	373	21.8 (19.9–23.8)	298	33	11.1 (7.7–15.2)
Total illiterate	369	181	49.1 (43.8–54.3)	95	21	22.1 (14.2–31.8)
Totals						
Grand total (unweighted)^a^	2110	562	26.6 (24.8–28.6)	408	59	14.5 (11.2–18.3)
Grand total (weighted)^b^	2229	602	27.9 (25.2–28.9)	432	54	12.5 (9.5–16.0)

The 10/66 short-form refers to the 10/66 Short Dementia Diagnostic Schedule.

a. Grand total may be higher than subgroups because of missing demographic details.

b. Weighted by national age, sex and literacy estimates; see Supplementary Appendix 3 for weightings.

### Associations with dementia

Across both countries, dementia increased with age and decreased in literate compared with illiterate participants. Dementia prevalence was lower in men compared with women in Indonesia, but no such association was found in South Africa. After adjusting for age, the associations remained largely unchanged in Indonesia, although illiteracy was no longer associated with dementia prevalence in South Africa (Table 3). Similar findings were found when calculating prevalence ratios (Supplementary Appendix 6).

**Table 3:** Odds of dementia against age, sex and literacy in Indonesia and South Africa

	Unadjusted odds ratio (95% CI)										Age-adjusted odds ratio	
	65–69 years^a^	70–74 years	75–79 years	80–84 years	85–89 years	≥90 years	Female^b^	Male	Illiterate^c^	Literate	Male	Literate
Indonesia	Reference	1.3 (1.0–1.6)	2.2 (1.6–2.9)	4.1 (2.8–5.9)	4.3 (2.3–8.1)	7.8 (2.3–26.1)	Reference	0.6 (0.5–0.7)	Reference	0.3 (0.2–0.4)	0.6 (0.5–0.8)	0.3 (0.2–0.4)
South Africa	Reference	9.5 (2.1–42.2)	6.4 (1.3–32.0)	15.6 (3.3–73.2)	44.8 (9.7–207.9)	38.7 (6.8–219.9)	Reference	1.0 (0.5–1.8)	Reference	0.4 (0.2–0.8)	1.3 (0.7–2.5)	0.5 (0.3–1.0)

a. Age compared with 65–69 year category.

b. Male compared with female category.

c. Literacy compared with illiterate category (unable to read or write).

### Concurrent validity

In both countries, the 10/66 short algorithm was able to differentiate scores based on the Brief CSI-D cognitive score, Brief CSI-D screening tool informant score, DSRS, Lawton Activities of Daily Living Scale and need for care (*P* < 0.001). All outcome variables had a large effect size between dementia positive and negative cases, with the exception of the need for care in Indonesia (Hedges' *g* = 0.70). The 10/66 short algorithm demonstrated good accuracy in Indonesia (AUC = 0.75, 95% CI 0.72–0.77) and very good accuracy in South Africa (AUC = 0.82, 95% CI = 0.76–0.88) against the DSRS screening cut-off. Similarly, the 10/66 algorithm demonstrated good accuracy in Indonesia (AUC = 0.79, 95% CI 0.76–0.81) and very good accuracy in South Africa (AUC = 0.80, 95% CI 0.73–0.87) against the Brief CSI-D screening tool (Supplementary Appendix 4).

## Discussion

This paper presents data on dementia prevalence from the STRiDE programme, serving as a proof of concept and validation of the STRiDE method for use in further studies in other LMICs. The data reported here applies standard methods, and contributes new, directly comparable, good-quality empirical data to the sparse dementia prevalence literature in two populous and culturally diverse middle-income countries, Indonesia and South Africa. This study is the first to generate prevalence data derived from the rural regions of North Sumatra (Indonesia) and Limpopo (South Africa). The findings indicate dementia prevalence estimates that are higher than those usually generated internationally, markedly so in the case of Indonesia. Our weighted prevalence estimates indicate that there may be 4 297 000 people with dementia in Indonesia and 450 000 people with dementia in South Africa (populations of people aged 65 years and older derived from 2018 estimates in Indonesia and 2020 estimates in South Africa; Supplementary Appendix 3). Our estimates exceed the numbers generated through modelling in the Global Burden of Disease 2019 study in Indonesia (768 000; 95% uncertainty interval 656 000–895 000) and South Africa (208 000; 95% uncertainty interval 179 000–241 000).^1^ The very low level of diagnosis of dementia in both countries is striking, with <1% of each sample reporting that they had received a diagnosis. Without diagnosis there is no chance of effective care and treatment for the person with dementia or support for their family carers. The results of this study illustrate the size of the challenge facing many countries and the importance of prioritising dementia at a policy level.

Although the estimates of dementia prevalence reported here look high, they may not be incorrect. The weighted dementia prevalence estimate for those aged 65 years and older in South Africa (12.5%) is in line with a previous study among isiXhosa speakers in Cape Town, which used the Brief CSI-D screening tool to identify cases (11%; 95% CI 9–13).^4^ Similarly, our prevalence estimate in Indonesia (27.9%) is in line with a growing evidence base across geographic regions in the country, albeit in those aged 60 years and older: Borobudur (15.9%),^6^ Yogyakarta (20.1%)^8^ and Jatinangor (29.2%).^7^ Some Indonesian studies have reported lower prevalence rates in certain settings: for example, dementia prevalence in Jakarta was estimated to be 4.5%,^6^ but the methodology in all of these studies is suboptimal and all previous studies used non-clinical diagnostic criteria or brief screening tools, which may introduce different and unquantified measurement bias than reported here (e.g. not accounting for depression as a comorbidity). The 10/66 Short Dementia Diagnostic Schedule does not require administration by clinicians and so has value in estimating prevalence in LMICs, not least because it is a more affordable strategy and does not require specialists to be diverted away from their clinical practice. Its validity has been extensively demonstrated across cultures and diverse settings.^12,16–18^

There are a number of potential explanations for the high prevalence rates found in this study compared with regional World Health Organization estimates. First, the 10/66 short algorithm generates variability in prevalence estimates depending on country, from 3.4% in rural China to 13.0% in the Dominican Republic.^12^ This country-specific variability is not dissimilar to the standard algorithm, but at present it does not appear that the short algorithm systematically overestimates prevalence compared with the standard algorithm. However, as with the standard algorithm,^24^ elevated prevalence may represent higher sensitivity enabling the detection of milder cases rather than generating false positives. Second, there is the question of education fairness. The 10/66 short schedule was developed to be more education-fair than DSM criteria,^24^ but reports have suggested the false positive rate of the 10/66 short algorithm in low education groups may be 5.5%.^18^ However, the exclusion of the illiterate subgroup from our analysis still yields prevalence rates higher than other international estimates of dementia. Third, the elevated prevalence could be real and explained by differences in risk factors in the populations studied. The comparatively higher prevalence in Indonesia could be driven, in part, by these population-level risk factors. For example, although Indonesia and South Africa both have a high cardiovascular disease burden,^25,26^ Indonesia has a higher prevalence of cerebrovascular disease.^27^ Selection bias and measurement error seem unlikely given our sampling and the fact that we rigorously translated and cross-culturally adapted the schedule,^3^ in addition to implementing robust, standardised procedures for data collection and management, including the training and close supervision of all researchers.

Our observed associations between dementia prevalence and sex, age and literacy are in line with previous evidence, which provides some validation of our findings. Both countries demonstrated the expected age-related trend: older subgroups had greater likelihood of having dementia compared with younger subgroups. As expected, literacy was protective of the likelihood of dementia in both countries, although the findings become non-significant after controlling for age in South Africa. The association between literacy and dementia prevalence can be explained in terms of cognitive reserve,^28^ with education increasing a person's cognitive reserve, thus delaying the clinical onset of the condition. Men were found to have reduced likelihood of dementia compared with women in Indonesia, but not in South Africa. Men are often reported to have a lower prevalence of dementia compared with women,^29^ which can be attributed to higher mortality, even within age groups, resulting from an accumulation of risk factors, such as increased risk of depression and cardiovascular disease.^30^ The fact that women were more likely to be illiterate than men in both countries provides additional complexity. If cognitive reserve is protective of dementia onset, this might demonstrate an important inequality that needs to be addressed, given that education increases cognitive reserve. Such late-life disadvantage in cognitive health in women as a result of inequality earlier in life has also been noted in other LMICs such as India.^31^

Strengths of our study include the use of a standardised toolkit and methodologies across two middle-income countries, harmonised in terms of outcome measures and derived using a good-quality, cross-cultural adaptation process. There are, however, important limitations to consider. First, data from South Africa must be considered as preliminary, as the sample is insufficiently powered and results in wide confidence intervals. The COVID-19 pandemic limited recruitment in South Africa, but the data are a proof of concept and allow for the design and delivery of a more definitive study. Anecdotally, the pandemic may also have led to selection bias owing to potentially vulnerable older adults being wary of face-to-face contact, even in the absence of governmental restrictions. Second, the sampling strategy was pragmatic and attempted to capture both rural and urban regions within each country. Although weighted prevalence estimates were calculated according to the national demographic profiles to improve generalisability, it is important to acknowledge that the heterogeneous nature of both countries’ populations increases the uncertainty of these estimates on a national level. The method could be used in other regions to generate more representative estimates at local and national levels. Third, our inclusion criteria may limit the generalisability of the findings. For example, it was necessary to have an informant (someone that knows the older adult well) so that the schedule could be completed. In North Sumatra, 20.7% of participants listed were ineligible, all of which were because they did not have an informant available to participate. This could mean that those who are the most socially isolated are not adequately represented in our sample. However, the fact that 11.5% of both cohorts included informants that were not friends, family members or neighbours could indicate that this group might still be represented. Finally, there is the possibility of instrument-related diagnostic error as discussed above. However, within the present study we had very good convergent validity. It was able to differentiate a series of cognitive, functional and care outcomes, in addition to having good discrimination ability against other estimates of dementia.

Our study provides novel, empirical evidence on the high numbers of people aged 65 years and older with dementia in Indonesia and South Africa, and the low level of current diagnosis in these communities. The findings are an improvement on existing estimates in terms of the quality of sampling and diagnostic methodology used. In adopting a robust yet pragmatic approach to estimating dementia prevalence, we present the STRiDE methodology that can be used within other LMIC settings in the future. There are also questions raised by the relatively high prevalence rates observed in this study compared with other international estimates, but even with this uncertainty, it is clear that dementia is common and should be accorded policy priority within each country. The fact that so few participants received a formal diagnosis highlights the size of the problem. Future research needs to explore how people's lives are affected by dementia within LMICs and the costs of care, particularly with the knowledge that health and social care systems are not sufficient to fully support people with dementia anywhere in the world.

## Data Availability

Data will become available on UK Data Services in 2023 and is available from the corresponding author, N.F.

## Supplementary material

For supplementary material accompanying this paper visit http://doi.org/10.1192/bjo.2023.76. click here to view supplementary material

## References

[1] GBD 2019 Dementia Forecasting Collaborators. Estimation of the global prevalence of dementia in 2019 and forecasted prevalence in 2050: an analysis for the Global Burden of Disease study 2019. Lancet Public Health 2022; 7(2): e105. 34998485 10.1016/S2468-2667(21)00249-8 PMC8810394

[2] Breuer E, Comas-Herrera A, Freeman E, Albanese E, Alladi S, Amour R, et al. Beyond the project: building a strategic theory of change to address dementia care, treatment and support gaps across seven middle-income countries. Dementia 2022; 21(1): 114–35. 34196585 10.1177/14713012211029105

[3] Farina N, Jacobs R, Sani TP, Schneider M, Theresia I, Turana Y, et al. Description of the cross-cultural process adopted in the STRiDE (STrengthening Responses to dementia in DEveloping countries) program: a methodological overview. Alzheimers Dement (Amst) 2022; 14(1): e12293. 35317433 10.1002/dad2.12293 PMC8923343

[4] de Jager CA, Msemburi W, Pepper K, Combrinck MI. Dementia prevalence in a rural region of South Africa: a cross-sectional community study. J Alzheimers Dis 2017; 60(3): 1087–96. 28984589 10.3233/JAD-170325 PMC5676974

[5] Prince M, Acosta D, Ferri CP, Guerra M, Huang Y, Jacob KS, et al. A brief dementia screener suitable for use by non-specialists in resource poor settings—the cross-cultural derivation and validation of the Brief Community Screening Instrument for Dementia. Int J Geriatr Psychiatry 2011; 26(9): 899–907. 21845592 10.1002/gps.2622 PMC3427892

[6] Ben-Arie O, Swartz L, Teggin AF, Elk R. The coloured elderly in cape town–a psychosocial, psychiatric and medical community survey. Part II. prevalence of psychiatric disorders. S Afr Med J 1983; 64(27): 1056–61. 6665654

[7] Hogervorst E, Mursjid F, Ismail RI, Prasetyo S, Nasrun M, Mochtar, et al. Validation of two short dementia screening tests in Indonesia. In Vascular Dementia: Risk Factors, Diagnosis and Treatment (ed S. R. Jacobsen): 235–56. Nova Science Publishers, 2011.

[8] Ong PA, Annisafitrie FR, Purnamasari N, Calista C, Sagita N, Sofiatin Y, et al. Dementia prevalence, comorbidities, and lifestyle among Jatinangor elders. Front Neurol 2021; 12: 643480. 34367043 10.3389/fneur.2021.643480 PMC8345013

[9] Suriastini N, Turana Y, Supraptilah B, Wicaksono TY, Mulyanto E. Prevalence and risk factors of dementia and caregiver's knowledge of the early symptoms of Alzheimer's disease. Aging Med Healthc 2020; 11: 60–6.

[10] Prince M, Wimo A, Guerchet M, Ali GC, Wu YT, Prina M. World Alzheimer Report 2015: The Global Impact of Dementia. Alzheimer's Disease International, 2015 (https://www.alzint.org/resource/world-alzheimer-report-2015/).

[11] Harris PA, Taylor R, Thielke R, Payne J, Gonzalez N, Conde JG. Research electronic data capture (REDCap)–a metadata-driven methodology and workflow process for providing translational research informatics support. J Biomed Inform 2009; 42(2): 377–81. 18929686 10.1016/j.jbi.2008.08.010 PMC2700030

[12] Stewart R, Guerchet M, Prince M. Development of a brief assessment and algorithm for ascertaining dementia in low-income and middle-income countries: the 10/66 short dementia diagnostic schedule. BMJ Open 2016; 6(5): e010712. 10.1136/bmjopen-2015-010712 PMC4885443 27225649

[13] Hall KS, Gao S, Emsley CL, Ogunniyi AO, Morgan O, Hendrie HC. Community screening interview for dementia (CSI ‘D’); performance in five disparate study sites. Int J Geriatr Psychiatry 2000; 15(6): 521–31. 10861918 10.1002/1099-1166(200006)15:6<521::aid-gps182>3.0.co;2-f

[14] Prince M, Reischies F, Beekman AT, Fuhrer R, Jonker C, Kivela SL, et al. Development of the EURO-D scale–a European, Union initiative to compare symptoms of depression in 14 European centres. Br J Psychiatry 1999; 174: 330–8. 10533552 10.1192/bjp.174.4.330

[15] Morris JC, Heyman A, Mohs RC, Hughes JP, van Belle G, Fillenbaum G, et al. The Consortium to Establish a Registry for Alzheimer's Disease (CERAD). Part I. Clinical and neuropsychological assessment of Alzheimer's disease. Neurology 1989; 39(9): 1159–65. 2771064 10.1212/wnl.39.9.1159

[16] Abdin E, Vaingankar JA, Picco L, Chua BY, Prince M, Chong SA, et al. Validation of the short version of the 10/66 dementia diagnosis in multiethnic Asian older adults in Singapore. BMC Geriatr 2017; 17: 94. 28431511 10.1186/s12877-017-0475-7 PMC5399400

[17] Ibnidris A, Piumatti G, Carlevaro F, Fadda M, Magno F, Magistro D, et al. Italian version of the short 10/66 dementia diagnostic schedule: a validation study. BMJ Open 2021; 11(6): e045867. 10.1136/bmjopen-2020-045867 PMC8246379 34193490

[18] Khan QUA, Prince MJ. Validation of the short version of the 10/66 dementia diagnosis in Urdu in Karachi, Pakistan. Alzheimer Dis Assoc Disord 2022; 36(1): 89–91. 34310442 10.1097/WAD.0000000000000467

[19] Clark CM, Ewbank DC. Performance of the Dementia Severity Rating Scale: a caregiver questionnaire for rating severity in Alzheimer disease. Alzheimer Dis Assoc Disord 1996; 10(1): 31–9. 8919494

[20] Moelter ST, Glenn MA, Xie SX, Chittams J, Clark CM, Marianne Watson I, et al. The Dementia Severity Rating Scale predicts clinical dementia rating sum of boxes scores. Alzheimer Dis Assoc Disord 2015; 29(2): 158–60. 24770371 10.1097/WAD.0000000000000031 PMC4208973

[21] Lawton MP, Brody EM. Assessment of older people: self-maintaining and instrumental activities of daily living. Gerontologist 1969; 9(3_Part_1): 179–86. 5349366

[22] Chandra V, Ganguli M, Pandav R, Johnston J, Belle S, DeKosky ST. Prevalence of Alzheimer's disease and other dementias in rural India: the Indo-US study. Neurology 1998; 51(4): 1000–8. 9781520 10.1212/wnl.51.4.1000

[23] Šimundić AM. Measures of diagnostic accuracy: basic definitions. EJIFCC 2009; 19(4): 203–11. 27683318 PMC4975285

[24] Prince M, Acosta D, Chiu H, Scazufca M, Varghese M. Dementia diagnosis in developing countries: a cross-cultural validation study. Lancet 2003; 361(9361): 909–7. 12648969 10.1016/S0140-6736(03)12772-9

[25] Chow C, Atkins E, Shariful Islam SM, Lung T. *Reducing the Burden of Cardiovascular Disease in Indonesia: Evidence Review*. The George Institute for Global Health, 2017 (10.13140/RG.2.2.20937.57445).

[26] Byrne J, Eksteen G, Crickmore C. Cardiovascular Disease Statistics Reference Document. The Heart and Stroke Foundation South Africa, 2016 (https://www.heartfoundation.co.za/wp-content/uploads/2017/10/CVD-Stats-Reference-Document-2016-FOR-MEDIA-1.pdf).

[27] Roth GA, Johnson C, Abajobir A, et al. Global, regional, and national burden of cardiovascular diseases for 10 causes, 1990 to 2015. J Am Coll Cardiol 2017; 70(1): 1–25. 28527533 10.1016/j.jacc.2017.04.052 PMC5491406

[28] Prince M, Acosta D, Ferri CP, Guerra M, Huang Y, Llibre Rodriguez JJ et al. Dementia incidence and mortality in middle-income countries, and associations with indicators of cognitive reserve: a 10/66 dementia research group population-based cohort study. Lancet 2012; 380(9836): 50–8. 22626851 10.1016/S0140-6736(12)60399-7 PMC3525981

[29] Eastman J, Bahorik A, Kornblith E, Xia F, Yaffe K. Sex differences in the risk of dementia in older veterans. J Gerontol A Biol Sci Med Sci 2022; 77(6): 1250–3. 35134198 10.1093/gerona/glac029 PMC9391887

[30] Li R, Singh M. Sex differences in cognitive impairment and Alzheimer's disease. Front Neuroendocrinol 2014; 35(3): 385–403. 24434111 10.1016/j.yfrne.2014.01.002 PMC4087048

[31] Angrisani M, Jain U, Lee J. Sex differences in cognitive health among older adults in India. J Am Geriatr Soc 2020; 68(S3): S20–8. 32815603 10.1111/jgs.16732 PMC7521343

