# Supplementary material for: Comprehensive measurement of the prevalence of dementia in low- and middle-income countries: STRiDE methodology and its application in Indonesia and South Africa
Source: BJPsych Open. 2023 Jun 6;9(4):e102. doi: 10.1192/bjo.2023.76 (PMC10305093; doi:10.1192/bjo.2023.76)
Supplement: Supplementary file 1 [file S2056472423000765sup001.docx]

SUPPLEMENTARY MATERIAL

Contents

[Appendix A: Sampling strategy 2](#_Toc111729295)

[Appendix B: Characteristics of key components of the 10/66 short shedule 4](#_Toc111729296)

[Appendix C: Weightings 5](#_Toc111729297)

[Appendix D: Demographics of participants split by age, sex and literacy. 7](#_Toc111729298)

[Appendix E: Poisson regression models for prevalence ratios 8](#_Toc111729299)

[Appendix F: Concurrent Validity 9](#_Toc111729300)

# Appendix A: Sampling strategy

**Country Context**

Indonesia is a lower-middle income country with a population of 270.20 million, of whom 11.35 million (4.2%) are aged 65 years and older. It has 34 provinces spread across over 17,000 islands. Indonesia has one official language (Bahasa Indonesia) and is predominantly Muslim. South Africa is an upper-middle income country of 60.14 million people, of whom 3.69 million (6.1%) are aged 65 years and over. South Africa has nine provinces and 11 official languages, featuring a myriad of cultures and traditions. It is known as one of the most unequal countries in the world, facing a triple challenge of high poverty, inequality and unemployment.

**Sampling strategy - Western Cape**

The Western Cape is a predominantly urban province and is the third most populace in South Africa. There is approximately 487,927 people aged 65 years and older. Approximately, 2.4% of adults have no schooling and 10% are below the food poverty line (unable to purchase or consume food). Sampling occurred within the urban centre of Cape Town. Wards were stratified according to low-, middle- and high-income strata and then randomly selected within each stratum proportional to size to identify 3 low, 3 middle and 2 high income wards across the 115 wards of the City of Cape Town.

Since the census data available is dated and likely to underestimate population size, researchers had to include a dwelling-counting and estimation exercise using aerial maps and walkabouts in each ward included in the survey. Maps were printed for each ward and divided into sub-areas for counting. The number of dwelling units were counted in each sub-area in a ward, including the number of units for apartment blocks and plots with multiple (often informal) household structures such as shacks, “wendy houses” or any kind of backyard dwelling used for living purposes. The total estimated number of dwellings for each ward was calculated and then divided by 50 (required number of households per ward) to obtain the interval between each dwelling to be selected. Based on the interval and the estimated total number of dwellings for each sub-area, the sampling distribution for each ward was calculated using a systematic random sampling, proportionate to population size (PPS) technique.

Eligible households were identified using a door-knocking survey approach, documenting the procedure by collecting data on (1) time, date, and location; (2) whether someone is home and if it is safe for field researchers to proceed; (3) who they spoke to and if there is a person living there that is 65 years and older; (4) willingness to participate and if so, (5) when would be a good time for the interview. If no one was home, the household would be revisited a maximum of 2 times before replacing with the household on the left (and then right) of the originally selected dwelling until eligible and willing participants were found. Data were also collected on the outcome of these door-knocking visits for each household visited during the survey, as well as reasons provided by residents for refusals. However, the application of the interval became problematic in more informal areas where the layout and infrastructure of plots and dwelling posed challenges in applying strict intervals. Also, for informal areas in particular, communities tend to be characterised by a younger population, migrant labour and clustering of older adults to certain areas within communities. These realities made it near impossible to identify eligible households applying the interval method. Therefore, for informal settlement areas the interval had to be relaxed. In these cases, the area was roughly divided into sub-areas (or zones) with field researcher pairs dropped at each of these zones. Field researchers would then walk down the road and ask community members about the community composition and to help identify persons 65 years and older living in the area. Where the interval was not successful, this flexibility still ensured a relatively equal distribution of sampling across the ward and sub-areas, while supporting the identification of eligible participants in relatively young, fluid communities. Where older adults lived in community clusters, field researchers ensured that participants were recruited with a minimum of a 50 household interval between them.

If there are multiple older adults residing within the household, the person whose birthday was next was selected.

**Sampling strategy - Limpopo Province**

Limpopo province is predominantly rural, land-locked region. There are 374,425 people 65 years and older. Approximately 13.9% have no schooling and 40% are under the food poverty line. Sampling occurred exclusively within 14 villages within the Dikgale locality. These villages are small and are part of a health research demographic surveillance site called DIMAMO, an ongoing partnership with the University of Limpopo (UL). Households are surveyed annually, with updated population information available for each dwelling across the 14 villages. These villages are homogenous in terms of socio-economic status and would give the study sample a representation relatively typical of the South African rural context.

The DIMAMO sampling frame was used to identify eligible households with participants who are 65 years and older. Households were selected using simple randomisation in selecting households with a member that is 65 years and over from the DIMAMO sampling frame for each of the 14 villages. In the event the selected household was unavailable, households would be replaced after 2 revisits. In the event of a refusal, the household was replaced by following the sample list of randomly selected households provided to each field research team.

**Sampling strategy – Jakarta and North Sumatra**

DKI Jakarta is a large urban area based on Java Island and is the capital of Indonesia. Jakarta has an estimated 530,102 people aged 65 years and older. North Sumatra in a large, predominantly rural province on the island of Sumatra. Medan is the largest city of the region. There are an estimated 144,998 people aged over the age of 65 years and older in the region.

In Indonesia, due to availability of census data, the sampling strategy was more consistent across sites. Sampling was done by multistage random sampling with the smallest unit in this study was the household. In the first stage, random sampling was conducted at the district level (DKI Jakarta- West Jakarta and North Jakarta; North Sumatera- Medan City and Serdang Bedagai District), the second stage at the urban village level, and the third stage at the household level. Sampling was based on PPS sampling.

A database of older adults living listed in the cadres’ registration were extracted (July-August 2021) for each village. These data were used as a sampling frame and the list was randomized. Older adults that were unreachable or refused to participate, the next older adult on the list were recruited. Only one older adult per household were recruited.

Appendix B: Characteristics of key components of the 10/66 short shedule

| Characteristics of key components of the 10/66 short schedule in Indonesia (n=2,110) and South Africa (n=408). | | | | | | | | | |
| --- | --- | --- | --- | --- | --- | --- | --- | --- | --- |
|  | Indonesia | | | |  | South Africa | | | |
|  | Missing (n) | Min | Max | Mean (SD) |  | Missing (n) | Min | Max | Mean (SD) |
| Cogscore | 0 | 2.06 | 37.89 | 27.82 (3.89) |  | 0 | 10.24 | 34.03 | 29.50 (3.70) |
| Relscore | 0 | 0.0 | 21.50 | 4.49 (3.67) |  | 0 | 0 | 18.5 | 2.81 (2.99) |
| EURO-D | 55* | 0 | 12 | 4.09 (2.56) |  | 24* | 0 | 10 | 3.64 (2.35) |
| *Missing less than 4 items, sufficient to run algorithm. | | | | | | | | | |

Appendix C: Weightings

For national weighting proportions (age, sex, literacy) were obtained from each country utilising the following criteria:

- Figures need to be derived from a reputable source
- A preference will be to select estimates from a single source to minimise heterogeneity in how they are derived.
- Identify estimates that utilise similar operational definitions as used in the present study.
- Estimates and proportions from older adults will be prioritised
- The most up-to-date estimates will be prioritised

Instances in which there are notable variations will be noted below.

| **Weightings used in South African cohort. National estimates used to calculate weightings were taken from Statistics South Africa (2021).** | | | |
| --- | --- | --- | --- |
| **Literacy*** | **Sex**** | **Age**** | **Weighting** |
| Literate | Male | 65-69 | 1.51864 |
| Literate | Male | 70-74 | 1.14669 |
| Literate | Male | 75-79 | 1.17252 |
| Literate | Male | 80+ | 0.76497 |
| Illiterate | Male | 65-69 | 1.24447 |
| Illiterate | Male | 70-74 | 0.93967 |
| Illiterate | Male | 75-79 | 0.96084 |
| Illiterate | Male | 80+ | 0.62687 |
| Literate | Female | 65-69 | 1.27024 |
| Literate | Female | 70-74 | 0.95913 |
| Literate | Female | 75-79 | 0.98073 |
| Literate | Female | 80+ | 0.63984 |
| Illiterate | Female | 65-69 | 1.04091 |
| Illiterate | Female | 70-74 | 0.78597 |
| Illiterate | Female | 75+ | 0.80367 |
| Illiterate | Female | 80+ | 0.52433 |
| *Functional literacy – ability to read and write with at least one language, aged 60 years and older, 2019 estimate.  **2020 mid-year population estimates | | | |

| **Weightings used in Indonesia cohort, sequentially derived (sex, literacy, age). National estimates used to calculate weightings were BPS-Statistics Indonesia (2019)** | | | |
| --- | --- | --- | --- |
| **Literacy*** | **Sex**** | **Age**** | **Weighting** |
| Literate | Male | 65-69 | 1.0056 |
| Literate | Male | 70-74 | 1.1299 |
| Literate | Male | 75+ | 1.6872 |
| Illiterate | Male | 65-69 | 0.7478 |
| Illiterate | Male | 70-74 | 0.8403 |
| Illiterate | Male | 75+ | 1.2547 |
| Literate | Female | 65-69 | 0.7985 |
| Literate | Female | 70-74 | 0.8972 |
| Literate | Female | 75+ | 1.3398 |
| Illiterate | Female | 65-69 | 0.5939 |
| Illiterate | Female | 70-74 | 0.6673 |
| Illiterate | Female | 75+ | 0.9964 |
| * Adults aged 50 years and older, 2018 estimates  ** 2018 estimate | | | |

Data sources used for weightings were also used to generate the population figures of people living with dementia based on weighted prevalence.

**References**

Statistics South Africa. (2021). *Marginalised Groups Series IV: The Social Profile of Older Persons, 2015–2019*. Statistics South Africa.

BPS-Statistics Indonesia. (2019). *Statistik Indonesia: Statistical Yearbook of Indonesia 2019*. BPS-Statistics Indonesia.

Appendix D: Concurrent Validity

| **Comparison of positive and negative cases of dementia (as determined by the 10/66 short algorithm) against established DSRS cut-offs.** | | | | |
| --- | --- | --- | --- | --- |
|  | **Indonesia** | | **South Africa** | |
|  | **10/66 short-form dementia diagnosis algorithm** | | **10/66 short-form dementia diagnosis algorithm** | |
|  | Negative cases | Positive cases | Negative cases | Positive cases |
| DSRS: negative cases | 1,124 (74.6%) | 139 (25.1%) | 261 (77.2%) | 8 (13.8%) |
| DSRS: positive cases | 382 (25.4%) | 414 (74.9%) | 77 (22.8%) | 50 (86.2%) |
| The threshold of DSRS <3 has previously been used to differentiate healthy controls with people with dementia (Roalf et al., 2013). DSRS = Dementia Severity Rating Scale | | | | |

| **Comparison of positive and negative cases of dementia (as determined by the 10/66 short algorithm) against established Brief CSI-D screening tool cut-offs.** | | | | |
| --- | --- | --- | --- | --- |
|  | **Indonesia** | | **South Africa** | |
|  | **10/66 short-form dementia diagnosis algorithm** | | **10/66 short-form dementia diagnosis algorithm** | |
|  | Negative cases | Positive cases | Negative cases | Positive cases |
| Brief CSI-D screen negative | 1,242 (80.2%) | 130 (23.1%) | 321 (92.0%) | 19 (32.2%) |
| Brief CSI-D screen positive | 306 (19.8%) | 432 (76.9%) | 28 (8.0%) | 40 (67.8%) |
| The threshold of Brief CSI-D < 5 has previously been used to screen for dementia. CSI-D = Community Screening Instrument for Dementia, | | | | |

**References:**

Prince, M., Acosta, D., Ferri, C. P., Guerra, M., Huang, Y., Jacob, K. S., Llibre Rodriguez, J. J., Salas, A., Sosa, A. L., Williams, J. D., & Hall, K. S. (2011). A brief dementia screener suitable for use by non-specialists in resource poor settings—The cross-cultural derivation and validation of the brief Community Screening Instrument for Dementia. International Journal of Geriatric Psychiatry, 26(9), 899–907. https://doi.org/10.1002/gps.2622

Roalf, D. R., Moberg, P. J., Xie, S. X., Wolk, D. A., Moelter, S. T., & Arnold, S. E. (2013). Comparative accuracies of two common screening instruments for the classification of Alzheimer’s disease, mild cognitive impairment and healthy aging. Alzheimer’s & Dementia : The Journal of the Alzheimer’s Association, 9(5), 529–537. https://doi.org/10.1016/j.jalz.2012.10.001

| **Differences between positive and negative cases of dementia (as determined by the 10/66 short algorithm) on cognitive performance, functional performance and care need outcomes.** | | | | | | |
| --- | --- | --- | --- | --- | --- | --- |
|  | **Indonesia** | | | **South Africa** | | |
|  | **10/66 short-form dementia diagnosis algorithm** | | | **10/66 short-form dementia diagnosis algorithm** | | |
|  | **Negative cases** | **Positive cases** | **Diff** | **Negative cases** | **Positive cases** | **Diff** |
|  | M (SD) | M(SD) | Effect size – Hedges *g* (95%CIs) | M (SD) | M(SD) | Effect size – Hedges *g* (95%CIs) |
| Brief CSI-D screening tool - Informant score (Higher = more impairment) | 1.2 (1.1) | 2.7 (1.4) | -1.32 (-1.42 to -1.21) | 0.9 (0.9) | 2.6 (1.3) | -1.76 (-2.06 to -1.45) |
| Brief CSI-D screening tool – cognitive score (Lower = more cognitive impairment) | 7.0 (1.3) | 5.7 (1.4) | 0.92 (0.82 to 1.02) | 7.8 (1.3) | 5.6 (1.9) | 1.59 (1.29 to -1.89) |
| DSRS (Higher = more impairment) | 1.8 (2.9) | 8.9 (8.8) | -1.35 (-1.46 to -1.25) | 1.7 (2.9) | 10.1 (8.2) | -2.03 (-2.34 to -1.71) |
| Lawton ADL scale (Lower = more impairment) | 5.5 (2.0) | 3.2 (2.3) | 1.12 (1.02 to 1.23) | 7.0 (1.4) | 4.3 (2.4) | 1.72 (1.42 to 2.02) |
|  | N (%) | N (%) | Effect size – Hedges *g* (95%CIs) | N (%) | N (%) | Effect – Hedges *g* (95%CIs) |
| Needs care: Occasionally or much of the time | 356 (23.0%) | 323 (57.9%) | 0.70 (0.61 to 0.78) | 101 (29.2%) | 51 (86.4%) | 0.92 (0.71 to 1.13) |
| ADL = Activities of Daily Living, CSI-D = Community Screening Instrument for Dementia, DSRS = Dementia Severity Rating Scale | | | | | | |

Appendix E: Demographics of participants split by age, sex and literacy.

| **Demographics of participants in each country split by age, sex and literacy (valid cases only).** | | | | | | | | | | | | | | |
| --- | --- | --- | --- | --- | --- | --- | --- | --- | --- | --- | --- | --- | --- | --- |
| **Indonesia** | | | | | | |  | **South Africa** | | | | | | |
|  | **Male** | | | **Female** | | |  | **Male** | | | **Female** | | | |
| **Age** | **Illiterate** | **Literate** | **All male** | **Illiterate** | **Literate** | **All female** |  | **Illiterate** | **Literate** | **All male** | **Illiterate** | **Literate** | **All female** |  |
| 65-69 | 49 (11.3%) | 386 (88.7%) | 435 | 111 (19.4%) | 461 (80.6%) | 572 |  | 7 (15.6%) | 38 (84.4%) | 45 | 17 (24.3%) | 53 (75.7%) | 70 |  |
| 70-74 | 22 (9.1%) | 219 (90.9%) | 241 | 73 (20.3%) | 286 (79.7%) | 359 |  | 6 (15.0%) | 34 (85.5%) | 40 | 15 (21.7%) | 54 (78.3%) | 69 |  |
| 75-79 | 11 (10.3%) | 96 (89.7%) | 107 | 53 (29.8%) | 125 (70.2%) | 178 |  | 3 (13.0%) | 20 (87.0%) | 23 | 14 (31.1%) | 31 (68.9%) | 45 |  |
| 80-84 | 5 (12.5%) | 35 (87.5%) | 40 | 24 (25.0%) | 72 (75.0%) | 96 |  | 2 (16.7%) | 10 (83.3%) | 12 | 10 (26.3%) | 28 (73.7%) | 38 |  |
| 85-89 | 1 (7.7%) | 12 (92.3%) | 13 | 13 (50.0%) | 13 (50.0%) | 26 |  | 3 (30.0%) | 7 (70.0%) | 10 | 11 (45.8%) | 13 (54.2%) | 24 |  |
| 90 plus | 0 (0.0%) | 2 (100.0%) | 2 | 7 (70.0%) | 3 (30.0%) | 10 |  | 0 (0.0%) | 3 (100.0%) | 3 | 6 (54.5%) | 5 (45.5%) | 11 |  |
| Total | 88 (10.5%) | 750 (89.5%) | 838 | 281 (22.6%) | 960 (77.4%) | 1,241 |  | 21 (6.8%) | 112 (93.2%) | 133 | 73 (28.4%) | 184 (71.6%) | 257 |  |

Appendix F: Poisson regression models for prevalence ratios

| **Prevalence ratios (95% Wald CIs) of dementia against age, sex and literacy in Indonesia and South Africa. Poisson regression models with robust variance.** | | | | | | | | | | | | | | | | |
| --- | --- | --- | --- | --- | --- | --- | --- | --- | --- | --- | --- | --- | --- | --- | --- | --- |
|  |  | |  |  | | **Prevalence ratio (95% CI)** | | | | | | | | **Age adjusted prevalence ratio** | | |
|  | | **65-69*** | | **70-74** | **75-79** | | **80-84** | **85-89** | **90 plus** | **Sex: Female**** | **Sex: Males** | **Illiterate***** | **Literate** | **Sex: Males** | **Literate** |  |
| Indonesia | | Ref | | 1.2 (1.0-1.5) | 1.8 (1.4-2.2) | | 2.5 (1.9 – 3.3) | 2.6 (1.6 – 4.0) | 3.3 (1.6 – 6.6) | Ref | 0.7 (0.6-0.8) | Ref | 0.4 (0.4-0.5) | 0.7 (0.6-0.8) | 0.5 (0.4-0.6) |  |
| South Africa | | Ref | | 8.3 (1.9 - 35.2) | 5.9 (1.3 - 27.6) | | 12.5 (2.9 - 54.3) | 25.7 (6.2 - 106.4) | 23.6 (5.2-106.6) | Ref | 1.0 (0.6-1.7) | Ref | 0.5 (0.3-0.8) | 1.2 (0.7-2.0) | 0.6 (0.4-1.0) |  |
| *Age in comparison to 65-69 category.  ** Male sex in comparison to female category  ***Literacy in comparison to illiterate category (unable to read or write) | | | | | | | | | | | | | | | | |
